# Thrombosis of portal, superior mesenteric, and splenic veins: a case report

**DOI:** 10.3389/fphar.2023.1246914

**Published:** 2023-09-06

**Authors:** N. Soghomonyan, H. Khachatryan, G. Soghomonyan, Q. Fleming

**Affiliations:** ^1^ Department of Vascular Surgery and Diabetic Foot, Yerevan Medical Center, Yerevan, Armenia; ^2^ Faculty of General Surgery, M. Heratsi State Medical University, Yerevan, Armenia; ^3^ Department of Physiology and Biophysics, Case Western Reserve University, Cleveland, OH, United States; ^4^ Department of Anesthesiology, The Ohio State University Wexner Medical Center, Columbus, OH, United States

**Keywords:** portal venous thrombosis (PVT), intestinal ischaemia, anticoagulation, deep vein thrombosis (DVT), systemic inflammation

## Abstract

Patients with venous thrombosis of splanchnic circulation represent a group of high risk with significant morbidity and mortality, if treatment is delayed. We present a patient with thrombosis of portal vein and its tributaries combined with deep venous thrombosis (DVT) of the lower extremities who was successfully treated with conservative management. This patient case highlights the importance of early empiric anti-inflammatory therapy along with systemic anticoagulation to reduce the intestinal inflammation and enteritis and break the vicious circuit resulting in secondary progressive thrombosis of the splanchnic veins, fluid shifts, and functional ileus. *Case presentation*: A previously healthy 61-years-old female patient with no significant medical history was admitted with progressive upper abdominal pain, nausea and vomiting, low-grade fever, mild signs of ileus, and malaise. Imaging studies revealed portal venous dilation reaching ∼20 mm with near-total obliteration of the lumen by a thrombus. In addition, thrombosis of superior mesenteric and splenic veins with thrombophlebitis was found. Imaging studies also confirmed the presence of DVT of lower extremities including thrombus propagation into the iliac veins. An immediate therapy was started with parenteral antibiotics, anti-inflammatory medications, systemic anticoagulants, and intravenous fluid infusions to restore the circulating volume deficit and treat electrolyte disbalance. With such therapy, the patient’s symptoms resolved within a month, and she was discharged from the hospital with full recovery. Heparin infusion was started to reach systemic anticoagulation. With resolution of symptoms, anticoagulation was continued with warfarin. We used non-steroidal anti-inflammatory drugs (NSAIDs) as a component in management of intestinal and systemic inflammation and multifocal thrombosis when the antiphospholipid syndrome was also on the list of differential diagnoses. *Conclusion*: We present a previously asymptomatic patient with progressive portal venous thrombosis and ascending DVT. Early establishment of diagnosis and initiation of therapy with systemic anticoagulants, anti-inflammatory and antibacterial drugs helped to stop thrombus progression, prevent irreversible intestinal ischemia, and allow for re-canalization of the occluded veins. This case highlights the importance of early interventions to improve the treatment outcome.

## Introduction

The interplay of chronic disease, inflammation and coagulation with thrombosis has always been in the focus of researchers and practitioners taking care of critically ill patients. If left untreated, these processes may eventually result in hemorrhagic and thrombotic complications, disseminated intravascular coagulation (DIC), and multi-organ failure with high morbidity and mortality (Yanuck et al., 2019). Thrombosis of portal vein and its tributaries is a condition requiring immediate action to reduce the risk of serious complications as splanchnic infarction and organ perforation (Sanyal et al., 2022). Rarely, this complication takes place without any history of significant disease in the past, or it may manifest as an ongoing inflammatory disease.

We present a patient, who was admitted with signs of lower extremity DVT, abdominal discomfort, and functional ileus. Aside from the DVT, further examination revealed splanchnic venous thrombosis with progressive abdominal complaints. Immediate conservative treatment was initiated with antimicrobial, anti-inflammatory, and anticoagulant therapy as well as intravenous fluid replacement to restore the volume deficit and eliminate electrolyte disbalance.

This case presentation highlights the importance of systemic approach to this complex pathological condition requiring early diagnosis and immediate intervention to stop thrombus progression and allow for time to restoration of the venous blood flow thus preventing bowel infarction and perforation. The therapeutic goals included early anticoagulation with anti-inflammatory and antimicrobial treatment to counteract thrombus formation, intestinal inflammation to break the pathophysiological circuits resulting in secondary progressive thrombosis of the splanchnic vessels: portal, superior mesenteric, and splenic veins.

## Case presentation

A previously healthy 61-years-old female patient with no significant past medical history was admitted with DVT, upper abdominal tenderness, nausea and vomiting, subfebrile fever, mild signs of ileus, and progressive malaise. The ultrasound examination revealed portal venous dilation with a diameter of 20 mm with a near-total occlusion of the lumen by intraluminal thrombi, presence of mild abdominal exsudation. The patient underwent an emergent abdominal computerized tomographic (CT) examination, which revealed thrombosis of portal, upper mesenteric, and splenic veins with small bubbles of gas located in the adipose tissue of the abdominal wall in the upper quadrant. Splenomegaly was excluded, but the splenic vein was dilated (11 mm) and entirely occluded by a longitudinal thrombotic mass with linear hypodense filling defect near the splenic hilum with a high probability of venous infarction. Identical changes were described in the superior mesenteric vein, the diameter of which reached 14 mm. Within the vein, there was a filling defect due to thrombotic occlusion with intraluminal thrombi involving larger venous mesenteric branches with a spread to smaller branches with adequate contrast filling. The remaining mesenteric and pancreatic veins were intact. There were no signs of cirrhosis or pancreatitis. The portal venous thrombi reached the intrahepatic venous branches including the bifurcation. The walls of all affected veins were markedly thickened, and overloaded portocaval anastomoses were observed. The patient’s clinical symptoms correlated with the imaging data and were suggestive of portal hypertension and thrombophlebitis. The laboratory tests correlated with an acute inflammatory reaction with elevation of erythrocyte sedimentation rate (46 mm/h), C-reactive protein (218 mg/L), and D-dimer (10.8 μg/mL).

An immediate treatment was started to control progression of splanchnic thrombosis and avoid life-threatening complications. The following treatment was initiated without delay:

✓ Intravenous hydration to restore the fluid deficit, treat the electrolyte imbalance, and maintain adequate diuresis.
✓ Systemic wide-spectrum antibacterial therapy was started with intravenous ceftriaxone, 2.0 g/day combined with Metronidazole, 500 mg, three times a day. Once the urine cultures came back positive for hemolytic Streptococci sensitive to Amoxicillin and Ciprofloxacin, further antibiotic therapy was continued with these drugs for additional 2 weeks.
✓ Non-steroidal anti-inflammatory therapy was initiated with Ibuprofen 400 mg three times a day.
✓ Systemic anticoagulation with intravenous infusion of unfractionated heparin with a goal to maintain the international normalized ratio (INR) between 2.0–3.0. After resolution of symptoms with restoration of venous blood flow verified by imaging studies, further anticoagulation was continued with oral Warfarin 5 mg for 6 months with periodic monitoring of the coagulation status.

With provided therapy, the patient’s symptoms gradually resolved, and the blood flow in splanchnic veins was re-established within a few days. In 3 days, once the clinical signs of ileus resolved, enteral feeding was started. The patient was discharged from the hospital with complete resolution of the symptoms. Her medical management was continued as an outpatient.

Serial clinical assessments, laboratory tests, ultrasound and CT imaging showed regression of portal hypertension and thrombophlebitis. Complete resolution of symptoms and return to normal daily activities became possible in 2 months after discharge from the hospital.

## Discussion

Thrombosis of portal and other veins in the splanchnic system, if not diagnosed and treated in time, carries the risk of intestinal ischemia with perforation of the affected portions of gut creating a life-threatening situation. Etiologically, portal thrombosis is more commonly seen in patients with liver cirrhosis, systemic pro-thrombotic conditions, malignancies, and several other conditions (Sanyal et al., 2022).

The clinical picture varies with acuity of the process and the extent of occlusion. The patients may be completely asymptomatic or may complain on abdominal tenderness, have signs of developing ileus, pancreatitis, variceal bleeding, etc. When mesenteric vessels are involved, the patients may develop a picture of overt mesenteric ischemia, infarction, peritonitis, and septic shock (Sanyal et al., 2022).

Early systemic anticoagulation to counteract the expansion of the venous thrombi and allow for recanalization of the major veins is the mainstay of therapy (Sanyal et al., 2022). It is also important to diagnose and treat the underlying conditions predisposing to venous thrombosis. Inflammatory bowel disease, infections, malignancy, systemic inflammatory reaction, antiphospholipid syndrome, pancreatitis, cirrhosis, hepato-portal sclerosis, and other conditions, including anatomical variants, may predispose or even trigger portal venous thrombosis (Beyazit et al., 2011; Talebi-Taher et al., 2018; Fan et al., 2019; Leitão et al., 2019). Our patient had no previous history of the above-mentioned pathologies, nevertheless, all of them were on our list of differential diagnoses. Laboratory tests and diagnostic studies were carried out to rule out autoimmune diseases, chronic infections, and malignancy. Since the patient presented with progressive signs of ileus and inflammation, systemic wide-spectrum antibiotics along with NSAIDs were given. Once the results of blood and urinary cultures became available indicating urinary infection with hemolytic Streptococci sensitive to Amoxicillin and Ciprofloxacin, further antimicrobial therapy was continued based on the microbial sensitivity. With improvement of symptoms and imaging evidence of partial recanalization of the portal and splanchnic venous blood flow, systemic anticoagulation was continued with warfarin.

While selecting the drug for chronic anticoagulation therapy, we considered the ample clinical experience and literature evidence on warfarin’s efficacy for patients with portal thrombosis and cirrhosis. While there are reports of potential efficacy of direct oral anticoagulants in patients with cirrhosis, there are also reports suggesting decreased efficacy of rivaroxaban and apixaban in those patients (Elhosseiny et al., 2019; Sanyal et al., 2022). There are also concerns for increased risk of bleeding, especially, when used with NSAIDs, which are used to treat local and systemic inflammatory processes predisposing to vascular thrombosis.

In addition to restoration of portal venous blood flow, therapeutic measures helped to control the progressive DVT with occlusion of the iliac veins. As in our patient, the management of DVT of the lower extremities and inferior vena cava is mainly conservative and includes systemic anticoagulation, compression stockings and treatment of complications (Talebi-Taher et al., 2018). Some patients will benefit from placement of an IVC filter to decrease the risk of thromboembolism. Invasive surgical interventions with successful endovascular thrombectomy, stenting, and prosthetic replacement of the inferior vena cava have also been reported, even though the procedures are not without risk (Gwozdz et al., 2018; Wei et al., 2018; Che et al., 2019; Mityul et al., 2019; Wagenhäuser et al., 2019; Yang et al., 2019).

At 5-month follow-up, the patient remained asymptomatic. She continued receiving anticoagulant therapy. She used compression stockings and postural measures for her DVT. Repetitive imaging confirmed partial recanalization of the iliac veins.

## Conclusion

Thrombosis of the portal vein and its branches is a serious complication and any delays in establishing the diagnosis and early intervention may be life-threatening. Pre-existing autoimmune disorders, malignancy, inflammatory bowel disease, infection, systemic inflammation with a surge in inflammatory cytokines should be considered as potential mechanisms for development of portal and splanchnic venous thrombosis. The time for diagnostic procedures should be minimized and therapeutic measures should be started early to avoid irreversible organ damage with increased morbidity and mortality. The presented patient case demonstrates that systemic anticoagulation along with anti-inflammatory and antimicrobial therapy, when an infectious etiology is suspected, helps to stop the thrombus progression, and allows for recanalization of the occluded veins. Timely interventions help to save lives and improve its quality.

## Data Availability

The raw data supporting the conclusion of this article will be made available by the authors, without undue reservation.
